# Physician estimates of inflammation, damage and distress in rheumatoid arthritis: how can they be used to improve patient care?

**DOI:** 10.1093/rap/rkae072

**Published:** 2024-06-05

**Authors:** Michaël Doumen, Elias De Meyst, Patrick Verschueren

**Affiliations:** Department of Development and Regeneration, KU Leuven, Leuven, Belgium; Department of Rheumatology, University Hospitals Leuven, Leuven, Belgium; Department of Development and Regeneration, KU Leuven, Leuven, Belgium; Department of Rheumatology, University Hospitals Leuven, Leuven, Belgium; Department of Development and Regeneration, KU Leuven, Leuven, Belgium; Department of Rheumatology, University Hospitals Leuven, Leuven, Belgium

**This editorial refers to ‘Physician estimate of inflammation vs global assessment in explaining variations in swollen joint counts in rheumatoid arthritis patients’ by Schmukler *et al*., 2024;https://doi.org/10.1093/rap/rkae057.**

In this issue, Schmukler *et al*. provide an interesting report on a physician estimate of inflammation and its ability to explain swollen joint counts (SJC) among patients with rheumatoid arthritis (RA) [1]. This report builds upon observations that the physician’s global assessment in RA (PhGA) is also affected by aspects not directly related to inflammation, including joint damage and comorbidities, which prompted the development of separate visual analogue scales for physician estimates of inflammation (DOCINF), damage (DOCDAM) and patient distress (DOCSTR). Schmukler *et al*. studied these scales’ correlations with measures of disease activity in routine care, concluding that DOCINF explained variations in SJC to a larger extent than the PhGA did.

The authors’ findings provide ample food for thought. First, it should be noted that a single assessor, who was aware of the study rationale, completed all scores and joint counts, which is expected to increase the scales’ specificity, in turn inflating the association between DOCINF and SJC. This also implies that information on inter-observer variability is currently lacking. Given that these physician estimates are specifically intended for use in daily practice, and thus often by various different physicians, the single assessor is an important limitation of this study affecting both the internal and external validity of the findings. However, we understand from the authors that studies with multiple assessors are currently ongoing, which will be informative. On the other hand, as corroborated by the authors’ commendable prior work, Schmukler *et al*.’s study confirms that DOCINF, DOCDAM and DOCSTR are not interchangeable and indeed provide additional insight to the PhGA. For instance, of the three scales, the PhGA correlated most strongly with DOCINF, but also with myriad other measures such as joint counts and patient-reported outcomes like pain and fatigue. This suggests that the PhGA is usually based on a combination of factors, which is consistent with prior literature [2], but is still mostly driven by the rheumatologists’ perception of inflammation. In turn, DOCINF was mainly explained by the SJC, DOCDAM by clinically deformed joints, and DOCSTR by patient-reported outcomes, particularly fatigue. Together, this suggests that rheumatologists indeed consider aspects like joint damage and fatigue as not directly related to active inflammation, while these do seem to affect their global consideration of disease control, the PhGA. Moreover, research has shown that the PhGA often differs substantially from the patient global assessment [3]. Perhaps, using distinct physician estimates of inflammation, damage and distress could thus help to mitigate discordance between patient and physician perceptions on disease activity or to facilitate patient–physician interaction about treatment targets [4]. In fact, the rationale behind these scales is reminiscent of the so-called ‘dual target’ strategy, which proposes to consider ‘biological remission’ (absence of disease activity) and the patient’s experience of disease control as separate targets which may require different interventions [5].

Despite the potential merits of the DOCINF, DOCDAM and DOCSTR measures, it is important to consider how their use should affect daily rheumatology practice. In their report, Schmukler *et al*. argue that most rheumatologists do not record formal joint counts in routine practice due to the additional time it requires to document these [6]. Consequently, the authors propose that DOCINF might serve as a practical alternative for a formal SJC. On the other hand, one could argue that DOCINF explained only 30% of SJC variation in their study and was itself equally driven by pain, which can have non-inflammatory or extra-articular causes. Perhaps more importantly though, while the positive impact of treating-to-target on patient outcomes in RA has been convincingly established, it is inherently impossible to apply this strategy without both performing and recording a formal tender and swollen joint count. As the authors highlight in their discussion, recording the joint count adds only about a minute to the consultation time [6]. One wonders if this amount of time saved is really worth foregoing treat-to-target principles. Indeed, recent literature shows that, despite the overwhelming evidence in its favour, the treat-to-target strategy is still not optimally implemented in daily practice [7]. Instead of looking for alternatives for a formal joint count, should we not invest our efforts into more effective implementation of a strategy that has clearly proven its worth?

A final point to consider is how physicians should approach the DOCSTR estimate. In their report, Schmukler *et al*. rightfully point out that all core clinical outcome measures for RA can be affected by comorbidities like fibromyalgia and depression, even in the absence of inflammatory disease activity. The authors then seemingly propose DOCSTR as a means to describe the influence of comorbidities on measures of RA disease activity. This raises a broader question: should all symptoms not directly related to inflammation be considered as caused by comorbidities? For instance, should feelings of anxiety or characteristics of central sensitisation necessarily imply an anxiety disorder or fibromyalgia? Although these are well-described and distinct conditions, health is more than the absence of morbidity. One could even argue that when living with RA, still a chronic condition characterised by pain, fatigue and unpredictably recurring flares, it is understandable to experience feelings of anxiety or depressed mood now and then. For example, prevalence estimates for depression among patients with RA are much higher when based on screening questionnaires, which can be affected by symptoms of RA itself, than when considering a formal diagnosis of clinical depression [8]. Of course, even then, depression remains more prevalent among patients with RA than in the general population, but this is true for many chronic diseases [9]. There is now ample evidence that RA disease activity and patient-reported outcomes like fatigue and psychosocial well-being affect each other in complex and bidirectional ways [10]. While it is of utmost importance to recognise true comorbidity when it is present, we believe persons living with RA should be approached more holistically than by designating all symptoms and experiences into either ‘inflammation’ or ‘comorbidity’. A physician estimate of patient distress can therefore certainly be informative, but we would envisage this as part of a holistic assessment rather than as a means to filter out comorbidities.

In summary, we congratulate Schmukler *et al*. and their research group with the extensive work to develop and validate these physician estimates of inflammation, damage and distress in RA. Although their latest report provides further illustration of the usefulness of these measures, we believe their added value does not lie in the potential to replace formal joint counts. Instead, we are convinced they could be used as tools to delineate reasons for persistent symptoms not directly related to remaining disease activity, to discuss these with the patient, and to decide upon the most suitable interventions to address these symptoms through shared decision-making.

## Data Availability

No new data were generated or analysed in support if this article.
